# Genome-Wide Analysis of Gene Expression in Primate Taste Buds Reveals Links to Diverse Processes

**DOI:** 10.1371/journal.pone.0006395

**Published:** 2009-07-28

**Authors:** Peter Hevezi, Bryan D. Moyer, Min Lu, Na Gao, Evan White, Fernando Echeverri, Dalia Kalabat, Hortensia Soto, Bianca Laita, Cherry Li, Shaoyang Anthony Yeh, Mark Zoller, Albert Zlotnik

**Affiliations:** Senomyx, Inc, San Diego, California, United States of America; The University of Queensland, Australia

## Abstract

Efforts to unravel the mechanisms underlying taste sensation (gustation) have largely focused on rodents. Here we present the first comprehensive characterization of gene expression in primate taste buds. Our findings reveal unique new insights into the biology of taste buds. We generated a taste bud gene expression database using laser capture microdissection (LCM) procured fungiform (FG) and circumvallate (CV) taste buds from primates. We also used LCM to collect the top and bottom portions of CV taste buds. Affymetrix genome wide arrays were used to analyze gene expression in all samples. Known taste receptors are preferentially expressed in the top portion of taste buds. Genes associated with the cell cycle and stem cells are preferentially expressed in the bottom portion of taste buds, suggesting that precursor cells are located there. Several chemokines including CXCL14 and CXCL8 are among the highest expressed genes in taste buds, indicating that immune system related processes are active in taste buds. Several genes expressed specifically in endocrine glands including growth hormone releasing hormone and its receptor are also strongly expressed in taste buds, suggesting a link between metabolism and taste. Cell type-specific expression of transcription factors and signaling molecules involved in cell fate, including KIT, reveals the taste bud as an active site of cell regeneration, differentiation, and development. IKBKAP, a gene mutated in familial dysautonomia, a disease that results in loss of taste buds, is expressed in taste cells that communicate with afferent nerve fibers via synaptic transmission. This database highlights the power of LCM coupled with transcriptional profiling to dissect the molecular composition of normal tissues, represents the most comprehensive molecular analysis of primate taste buds to date, and provides a foundation for further studies in diverse aspects of taste biology.

## Introduction

Taste is fundamental for the selection of nutritious foods and rejection of poisonous or harmful substances [1]. In addition, taste plays a significant role in the hedonistic aspect of feeding. Loss of taste negatively impacts well being and is a significant morbidity factor in patients undergoing chemotherapy and radiation therapy [2]. The mouth contains thousands of specialized sensory taste buds. Each taste bud is made up of 50-100 cells classified historically by morphology and histology staining patterns into type I, II and III cells [3]. While less is known about the function(s) of type I cells, type II cells detect sweet, bitter and umami tastants via G protein-coupled receptors and type III detect sour tastants via ion channels [4], [5].

Characterization of gene expression in mammalian taste buds has largely been limited to rodents. Here, we report the results of a systematic and comprehensive survey of gene expression in taste buds isolated from a primate, the cynomolgus macaque (*Macaca fascicularis*). A close relative to humans that diverged 25 million years ago, the macaque represents a model system for human physiology, prefers a similar omnivorous diet, and shares an overall 93% genomic sequence identity (97.5% identity in orthologous genes). Sequencing of the macaque genome was recently completed [6] and used to construct a microarray based on the Human Genome U133 Plus 2.0 Array, thereby enabling expression analysis of over 47,000 macaque transcripts.

We report the identification of over 2,300 taste bud-associated genes, the majority of which have not been described previously in taste tissue, and numerous processes and pathways active in primate taste buds. The taste bud gene expression database forms the basis of more detailed studies to further explore taste biology in primates as well as humans.

## Results

### Generation of Primate Taste Bud Gene Expression Database

**Figure 1:**
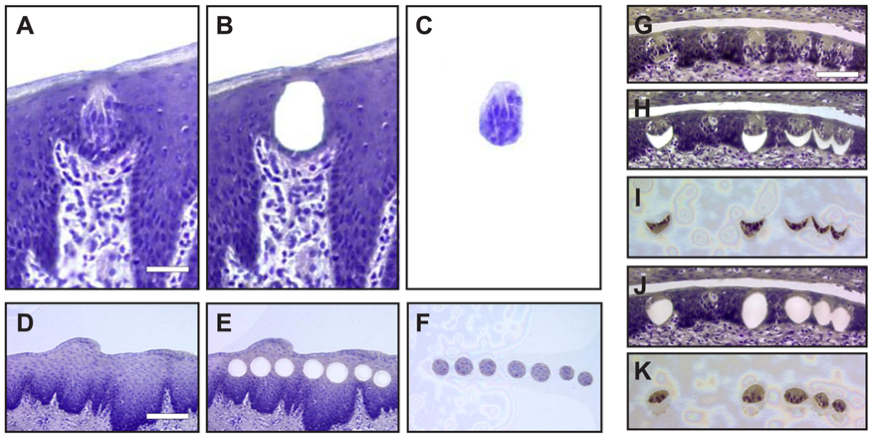
LCM of macaque taste tissue. Intact FG papilla section (A), residual tissue after LCM (B), and isolated FG taste bud area (C). Intact LE section (adjacent to FG papilla) (D), residual tissue after LCM (E), and isolated LE areas (F). (G–K) Collection of top and bottom TB fractions by LCM. Intact CV papilla section (G), section with bottom fraction removed (H), isolated bottom fraction (I), section with top fraction removed (J), and isolated top fraction (K). Scale bar is 20 µm in A and represents panels A–C, 40 µm in D and represents panels D–F, and 40 µm in G and represents panels G–K.

We used laser capture microdissection (LCM) [7], [8] to collect taste buds from fungiform (FG) papilla on the anterior tongue and circumvallate (CV) papilla on the posterior tongue of macaques (Figure 1). In FG papilla, one to three taste buds were observed immediately beneath the keratinized lingual surface whereas in CV papilla, numerous taste buds were observed along the inner walls of the clefts perpendicular to the lingual surface. Taste buds were readily identifiable in all sections used to collect samples and we estimate that the collected taste bud areas contained over 95% taste cells. Equivalent numbers of lingual epithelial cell areas were collected from surface epithelium immediately surrounding both FG and CV papilla (Figure 1D-F).

To identify taste bud-associated genes, we compared gene expression between isolated taste buds and lingual epithelium lacking taste buds. Since taste cells are specialized neuroepithelial cells, we focused on genes expressed at significantly higher levels in taste buds versus adjacent non-gustatory lingual epithelium. This allowed us to eliminate genes expressed in both locations and to identify genes involved in pathways and processes specific to taste.

In the first set of microarray experiments, we generated gene expression data from ten lingual epithelium (LE) samples and ten taste bud (TB) samples - six from FG papilla and four from CV papilla. All CV samples were collected from macaques also used to generate FG samples. We collected 4,700–6,400 estimated cells per sample that yielded sufficient total RNA for use in our GeneChip analyses (see Table S1). Cell numbers were calculated by counting cells in each taste bud area and multiplying by the number of taste buds collected. The resulting expression data files (.cel files) were uploaded to GeneSpring GX software for analysis. Features were background corrected and quantile normalized using GC-RMA [9], and GC-RMA normalized data were used to identify TB-associated genes.

During histological analyses of a set of TB-associated genes identified from this first microarray set, we discovered that certain transcripts were specifically enriched in cells located at the bottom of the taste bud, suggesting divergent gene expression between the top and bottom portions of taste buds. This observation prompted us to collect a second set of LCM samples isolated from either the top (n = 4) or bottom (n = 4) fractions of CV TB (Figure 1G–K). These samples (n = 8) were obtained from different macaques than those from the first experiment and processed separately for microarray analyses (Table S1). Gene expression data were combined with the first set of 20 samples to yield a 28 sample data set. We re-normalized all 28 samples using the GC-RMA algorithm. All 28 samples passed data quality control parameters with a mean +/− SD % present call of 41±9%. Principal component analysis showed three clusters: LE samples, FG TB and CV TB samples from the first microarray set, and top and bottom CV TB samples from the second microarray set (clustered adjacent to the whole TB samples) (Figure S1). These observations are consistent with distinct patterns of gene expression between TB and LE samples.

TB-associated genes were identified in a multi-step process. First, we eliminated poorly performing probe sets (those with a mean intensity ≤25 in TB samples). Then using a series of pairwise comparisons between different sample sets we calculated fold change and p values (Student's *t*-test). The pairwise comparisons were: CV TB versus LE, FG TB versus LE, CV TB top versus LE, and CV TB bottom versus LE. Finally we selected probe sets representing genes significantly expressed at specific sites using the following inclusion values: expression ratio ≥5-fold, p value ≤0.05 to generate a TB-associated probe set list. The list of TB-associated probe sets was then extensively annotated by reference to the human genome and RefSeq database. Database annotation included: probe set ID, gene title, gene symbol, pairwise comparisons with corresponding p-values, known or predicted location of gene product, and functional classification of gene product. Finally, redundant probe sets were removed resulting in a database that contained 2,382 TB-associated genes. The complete TB-associated gene database is shown in Table S2.

### Expression of Taste Receptor Genes

Humans have ∼30 known taste receptor/candidate taste receptor genes: TAS1R1 (umami), TAS1R2 (sweet), TAS1R3 (umami and sweet co-receptor); PKD2L1 and PKD1L3 (candidate sour), and 25 TAS2Rs (bitter). The Affymetrix Rhesus macaque array contains probe sets corresponding to 26 of these genes, summarized by their TB versus LE expression ratios in Table 1. We observed higher expression of TAS1R1 and TAS1R2 in FG TB than in CV TB. In contrast, expression of most TAS2R genes was higher in CV TB compared to FG TB with TAS2R13 exhibiting the highest differential.

**Table 1:** Gene expression data for known taste receptor genes.

GENE TITLE	GENE SYMBOL	Type	Ratio CV vs. LE	p value CV vs. LE	Ratio FG vs. LE	p value FG vs. LE	Ratio CV_T vs. CV_B	p value CV_T vs. CV_B
polycystic kidney disease 1-like 3	PKD1L3	Sour	ND	ND	ND	ND	ND	ND
polycystic kidney disease 2-like 1	PKD2L1	Sour	16.95	0.0075	9.45	0.0012	2.85	0.0115
taste receptor, type 1, member 1	TAS1R1	Umami	1.95	0.1510	11.84	0.0000	2.73	0.0848
taste receptor, type 1, member 2	TAS1R2	Sweet	1.73	0.1269	4.46	0.0115	3.72	0.0387
taste receptor, type 1, member 3	TAS1R3	Co-receptor	ND	ND	ND	ND	ND	ND
taste receptor, type 2, member 1	TAS2R1	Bitter	1.22	0.5092	0.77	0.0425	1.24	0.0572
taste receptor, type 2, member 3	TAS2R3	Bitter	0.97	0.8095	1.00	0.9444	1.24	0.2648
taste receptor, type 2, member 4	TAS2R4	Bitter	41.63	0.0134	34.88	0.0008	1.77	0.0321
taste receptor, type 2, member 5	TAS2R5	Bitter	0.80	0.0787	0.75	0.0121	1.55	0.1448
taste receptor, type 2, member 7	TAS2R7	Bitter	7.90	0.3310	1.91	0.0498	4.22	0.0673
taste receptor, type 2, member 8	TAS2R8	Bitter	31.05	0.0511	7.18	0.0068	5.28	0.0059
taste receptor, type 2, member 9	TAS2R9	Bitter	1.06	0.6292	1.15	0.4684	1.46	0.3786
taste receptor, type 2, member 10	TAS2R10	Bitter	30.76	0.0044	20.29	0.0122	6.01	0.0310
taste receptor, type 2, member 13	TAS2R13	Bitter	25.56	0.1093	1.05	0.7620	8.50	0.0792
taste receptor, type 2, member 14	TAS2R14	Bitter	609.11	0.0010	135.57	0.0523	2.14	0.0035
taste receptor, type 2, member 16	TAS2R16	Bitter	4.77	0.1754	2.75	0.0345	8.76	0.0159
taste receptor, type 2, member 38	TAS2R38	Bitter	0.81	0.1508	0.86	0.2737	1.46	0.1289
taste receptor, type 2, member 39	TAS2R39	Bitter	5.56	0.2110	2.03	0.0899	3.94	0.0003
taste receptor, type 2, member 40	TAS2R40	Bitter	1.11	0.5487	1.31	0.1024	0.99	0.9819
taste receptor, type 2, member 41	TAS2R41	Bitter	1.10	0.7175	1.14	0.4309	1.33	0.1423
taste receptor, type 2, member 42	TAS2R42	Bitter	151.50	0.0019	107.25	0.0005	3.48	0.0003
taste receptor, type 2, member 43	TAS2R43	Bitter	14.78	0.0445	2.19	0.1445	5.89	0.0054
taste receptor, type 2, member 44	TAS2R44	Bitter	1.34	0.0292	1.40	0.0013	2.63	0.1855
taste receptor, type 2, member 45	TAS2R45	Bitter	ND	ND	ND	ND	ND	ND
taste receptor, type 2, member 46	TAS2R46	Bitter	3.70	0.0211	1.53	0.0485	0.92	0.7568
taste receptor, type 2, member 47	TAS2R47	Bitter	ND	ND	ND	ND	ND	ND
taste receptor, type 2, member 48	TAS2R48	Bitter	27.42	0.0062	11.27	0.0129	2.32	0.0879
taste receptor, type 2, member 49	TAS2R49	Bitter	5.02	0.3327	1.22	0.5023	6.49	0.0003
taste receptor, type 2, member 50	TAS2R50	Bitter	15.40	0.0946	20.53	0.0045	4.59	0.0616
taste receptor, type 2, member 60	TAS2R60	Bitter	3.36	0.4111	1.33	0.3660	1.15	0.6358

Average expression values from circumvallate taste bud (CV), fungiform taste bud (FG), CV TB top fraction (CV_T), CV TB bottom fraction (CV_B), and non-gustatory LE (LE) samples were used to calculate expression ratios. Corresponding p values were generated using two-sample paired t-tests. TAS1R3 is a co-receptor with TAS1R1 or TAS1R2; ND, not determined (gene not represented on array).

### Gene Expression Differences in Top and Bottom TB Fractions

We sought to identify genes expressed exclusively or predominantly in the bottom or top portions of CV TB. A total of 159 TB-associated genes exhibited intra-TB expression differences (≥5-fold CV TB bottom versus CV TB top ratio or ≥5-fold CV TB top versus CV TB bottom ratio, p value ≤0.05): 79 genes in the CV TB bottom fraction (Table S3) and 80 genes in CV TB top fraction (Table S4). The most abundant functional class of genes in the CV TB bottom fraction encoded extracellular matrix (ECM) proteins (n = 18), indicating that basal taste bud cells may actively secrete matrix components and participate in basement membrane formation. The next most abundant functional class was cell cycle genes (n = 12), indicating active cell division at the bottom of the taste bud. In contrast, only two ECM genes and no cell cycle genes were identified in the CV TB top fraction. Instead, the CV TB top fraction preferentially expressed many genes associated with signaling (7 receptors and 4 signaling components), metabolic processes (13 enzymes), and, importantly, taste (5 taste receptors). A comparison of expression of all taste receptor genes represented on the array indicates that taste receptor genes are expressed at higher levels in the top fraction with a mean top versus bottom ratio of 3.3 (Table 1). We propose that expression of novel taste receptor genes will also follow this pattern and be enriched in the top fraction of taste buds. Indeed, these data allowed us to identify a set of novel taste bud-associated genes that may encode taste receptors.

### The Top 50 Taste Bud-Associated Genes

Genes with the highest differential expression in TB relative to LE were identified. Tables 2 and 3 list the 50 top genes relative to either CV (Table 2) or FG samples (Table 3). Six genes known to be involved in taste signaling are included in this list: two taste receptors (TAS2R14 and TAS2R42), three heterotrimeric G protein subunits (GNAT3, GNB3, and GNG13), and a phospholipase (PLCB2) all of which participate in sweet, bitter, and umami taste signaling [10], [11]. Surprisingly, the top taste bud associated gene encodes a chemokine, CXCL14. Two additional chemokines, CXCL8 and CCL2, as well as a cytokine, TGFB2, are also present suggesting a role of immune-associated pathways in the taste buds. However, the largest functional group represented comprises neuron-associated genes (10 examples). Stem cell and developmental genes also figure prominently (5 genes) indicating that the taste bud is a site of active cell growth and differentiation. The single growth factor gene, SHH, has been linked to taste bud renewal and is preferentially expressed at the base of taste buds [3], [12,].

**Table 2:** Top twenty five circumvallate (CV) TB-associated genes.

Gene Title	Gene Symbol	Ratio CV vs. LE	Ratio FG vs. LE	Location	Function
chemokine (C-X-C motif) ligand 14	CXCL14	791	688	Secreted	Immune
taste receptor, type 2, member 14	TAS2R14	609	136	TM	Taste
solute carrier family 35, member F1	SLC35F1	555	335	TM	Transporter
Phosphodiesterase 1C, calmodulin-dependent 70kDa	PDE1C	407	259	Intracellular	Enzyme
matrix metallopeptidase 7 (matrilysin, uterine)	MMP7	348	94	Secreted	Enzyme
GDNF family receptor alpha 3	GFRA3	283	128	GPI	Neuronal
advillin	AVIL	254	237	Intracellular	Neuronal
zinc finger protein 483	ZNF483	241	173	Nuclear	Transcription factor
potassium voltage-gated channel, Isk-related family, member 3	KCNE3	210	208	TM	Channel
phospholipase C, delta 4	PLCD4	199	150	Intracellular	Signaling
calpain 9	CAPN9	187	72	Secreted	Enzyme
achaete-scute complex-like 1	ASCL1	176	155	Nuclear	Neuronal
Hypothetical protein LOC644139	LOC644139	173	139	TM	Multi-TM
collagen, type IX, alpha 2	COL9A2	168	92	Intracellular	Structural
insulin-like growth factor 1 (somatomedin C)	IGF1	167	66	Secreted	Growth factor
carboxypeptidase E	CPE	166	155	Intracellular	Enzyme
guanine nucleotide binding protein (G protein), gamma 13	GNG13	165	160	Intracellular	Taste
protein tyrosine phosphatase, receptor type, D	PTPRD	162	86	TM	Neuronal
taste receptor, type 2, member 42	TAS2R42	152	107	TM	Taste
hypothetical protein LOC253012	LOC253012	136	89	TM	Unknown
TOX high mobility group box family member 3	TOX3	135	134	Nuclear	DNA binding
weakly similar to XP_518970.1 similar to GLCCI1 protein	Hs.164557	135	12	N/A	Non-coding
tubulin, beta 2B	TUBB2B	133	101	Intracellular	Structural
espin	ESPN	127	106	Intracellular	Sensory
phospholipase C, beta 2	PLCB2	120	88	Intracellular	Taste

Genes are ranked by taste bud/non-gustatory lingual epithelium (LE) gene expression ratio. Average expression values from CV taste bud (CV), fungiform taste bud (FG), and LE samples were used to calculate expression ratios. Corresponding p values were generated using two-sample paired Student's t-tests. Location; known or predicted location of gene product, TM; known to be membrane-associated or with a predicted transmembrane domain, GPI; glycosylphosphatidylinositol-linked. Function; known or predicted function of gene product.

**Table 3:** Top twenty five fungiform (FG) TB-associated genes.

Gene Title	Gene Symbol	Ratio CV vs LE	Ratio FG vs LE	Location	Function
guanine nucleotide binding protein, alpha transducing 3 (gustducin)	GNAT3	344	594	Intracellular	Taste
SATB homeobox 2	SATB2	351	461	Nuclear	Development
multiple C2 domains, transmembrane 1	MCTP1	362	362	TM	Multi-TM
sodium channel, voltage-gated, type III, alpha	SCN3A	166	362	TM	Channel
transforming growth factor, beta 2	TGFB2	298	338	Secreted	Immune
doublecortin-like kinase 1	DCLK1	175	322	Intracellular	Structural
secretogranin V (7B2 protein)	SCG5	232	269	Intracellular	Neuronal
keratin 20	KRT20	115	252	Intracellular	Structural
interleukin 8	IL8	51	247	Secreted	Immune
Prospero-related homeobox 1	PROX1	86	228	Nuclear	Stem cell
insulinoma-associated 1	INSM1	96	223	Nuclear	Stem cell
synaptotagmin I	SYT1	169	208	Vesicular	Neuronal
neuronal cell adhesion molecule	NRCAM	130	196	TM	Neuronal
Seizure related 6 homolog (mouse)-like	SEZ6L	1	182	TM	Cell adhesion
KIAA1324	KIAA1324	138	159	TM	Unknown
stearoyl-CoA desaturase 5	SCD5	115	158	Intracellular	Enzyme
sema domain, immunoglobulin domain (Ig), short basic domain, sec, ted, (semaphorin) 3D	SEMA3D	13	150	TM	Stem cell
transmembrane protein 163	TMEM163	98	148	TM	Multi-TM
cadherin 2, type 1	CDH2	125	146	TM	Neuronal
neurexin 3	NRXN3	141	146	TM	Neuronal
chemokine (C-C motif) ligand 2	CCL2	9	132	Secreted	Immune
Sonic hedgehog homolog (Drosophila)	SHH	80	130	Secreted	Stem cell
BMP and activin membrane-bound inhibitor homolog	BAMBI	83	122	TM	Receptor
janus kinase and microtubule interacting protein 2	JAKMIP2	94	122	Intracellular	Neuronal
guanine nucleotide binding protein (G protein), beta polypeptide 3	GNB3	103	105	Intracellular	Taste

Genes are ranked by taste bud/non-gustatory lingual epithelium (LE) gene expression ratio. Average expression values from CV taste bud (CV), fungiform taste bud (FG), and LE samples were used to calculate expression ratios. Corresponding p values were generated using two-sample paired Student's t-tests. Location; known or predicted location of gene product, TM; known to be membrane-associated or with a predicted transmembrane domain. Function; known or predicted function of gene product.

### Region-Specific Taste Gene Expression

Next, we identified genes expressed exclusively or predominantly in CV but not FG taste buds and vice versa. A total of 54 TB-associated genes were site-specifically expressed (≥5-fold TB versus LE ratio, p value ≤0.05 AND ≥5-fold CV versus FG ratio or FG versus CV ratio): 23 genes in CV TB (Table 4) and 31 genes in FG TB (Table 5). A protein with protease inhibitor activity, sparc/osteonectin, cwcv and kazal-like domains proteoglycan (testican) 1 (SPOCK1), tops the CV-specific list. SPOCK1 is expressed predominantly in the brain and at lower levels at other sites including endothelial cells and the eye but its function(s) at these other sites is less well understood [13], [14]. Other CV-specific genes of note include the protein tyrosine kinase receptor ERBB4, the cholinergic receptor CHRNA10, and IGF1, a growth factor linked to growth hormone activity [15]. Seizure 6 like (SEZ6L) tops the FG-specific list. SEZ6L encodes an adhesion-like type I membrane protein with unknown function associated with lung and gastric cancer [16], [17]. The FG-specific list also includes receptors (HTR3E, EFNB3), immune-associated genes (IL1B, CXCL3, ICAM1, CD274), and secreted molecules (PTHLH, FGF19). Site-specific gene expression suggests that taste bud function varies by location on the tongue and that taste cell signaling and modulation can occur in a site-specific manner.

**Table 4:** Gene products predicted to be enriched in circumvallate (CV) TBs.

GENE TITLE	GENE SYMBOL	Ratio CV vs. FG	Ratio CV vs. LE	Ratio FG vs. LE	Location	Function
sparc/osteonectin, cwcv and kazal-like domains proteoglycan (testican) 1	SPOCK1	79.96	20.28	0.25	Secreted	Protease inhibitor
v-erb-a erythroblastic leukemia viral oncogene homolog 4 (avian)	ERBB4	31.71	57.07	1.80	TM	Receptor
SAM domain containing 5	SAMD5	25.60	31.68	1.24	Intracellular	Signaling
cholinergic receptor, nicotinic, alpha polypeptide 10	CHRNA10	24.58	18.13	0.74	Intracellular	Receptor
estrogen-related receptor gamma	ESRRG	21.24	49.03	2.31	Nuclear	Receptor
dopa decarboxylase (aromatic L-amino acid decarboxylase)	DDC	18.84	43.68	2.32	Intracellular	Enzyme
solute carrier family 1 (glial high affinity glutamate transport, ), member 3	SLC1A3	17.42	11.65	0.67	TM	Carrier
solute carrier family 26, member 7	SLC26A7	13.15	99.68	7.58	TM	Carrier
family with sequence similarity 46, member C	FAM46C	12.58	16.69	1.33	Intracellular	Unknown
histone deacetylase 9	HDAC9	11.94	30.12	2.52	Nuclear	Transcription factor
potassium channel, subfamily T, member 2	KCNT2	10.13	47.31	4.67	TM	Channel
Solute carrier family 24, member 5	SLC24A5	9.80	12.51	1.28	TM	Carrier
homeobox B3	HOXB3	8.63	46.71	5.41	Nuclear	Transcription factor
far upstream element (FUSE) binding protein 1	FUBP1	8.40	11.65	1.39	Nuclear	Gene expression
transmembrane 4 superfamily member 2	TM4SF2	6.89	65.32	9.48	TM	Unknown
MRNA; cDNA DKFZp686B0610 (from clone DKFZp686B0610)	AL832122	5.98	10.42	1.74	N/A	Unknown
poly(A) binding protein, cytoplasmic 5	PABPC5	5.93	14.69	2.48	Intracellular	RNA binding
insulin-like growth factor 1 (somatomedin C)	IGF1	5.82	39.61	6.81	Secreted	Growth factor
growth factor receptor-bound protein 14	GRB14	5.68	32.54	5.73	Intracellular	Signaling
cysteine dioxygenase, type I	CDO1	5.49	75.96	13.83	Intracellular	Enzyme
thrombospondin 4	THBS4	5.12	50.22	9.82	Secreted	ECM
CKLF-like MARVEL transmembrane domain containing 2	CMTM2	5.03	100.21	19.93	TM	Unknown
stanniocalcin 1	STC1	5.01	20.46	4.08	Secreted	Hormone

Genes with a CV versus fungiform (FG) TB expression ratio ≥5-fold are listed. Expression ratios of CV or FG TB versus lingual epithelium (LE) samples are also included. Location; known or predicted location of gene product, TM; known to be membrane-associated or with a predicted transmembrane domain, N/A; not applicable. Function; known or predicted function of gene product.

**Table 5:** Gene products predicted to be enriched in fungiform (FG) TBs.

GENE TITLE	GENE SYMBOL	Ratio FG vs. CV	Ratio CV vs. LE	Ratio FG vs. LE	Location	Function
Seizure related 6 homolog (mouse)-like	SEZ6L	217.50	0.84	182.37	TM	Cell adhesion
Elastin microfibril interfacer 2	EMILIN2	32.46	0.79	25.66	Secreted	ECM
parathyroid hormone-like hormone	PTHLH	23.47	2.00	47.01	Secreted	Hormone
5-hydroxytryptamine receptor 3 subunit E	HTR3E	20.62	1.04	21.48	TM	Receptor
regulator of G-protein signalling 4	RGS4	19.34	0.99	19.10	Intracellular	Signaling
polycystic kidney and hepatic disease 1 (autosomal recessive)	PKHD1	18.55	1.74	32.29	TM	Signaling
Unc-5 homolog C (C. elegans)-like	UNC5CL	18.19	3.83	69.73	Intracellular	Signaling
multiple EGF-like-domains 10	MEGF10	17.77	1.39	24.75	TM	Unknown
ubiquitin D	UBD	15.15	2.97	45.00	Intracellular	Apoptosis
chemokine (C-C motif) ligand 2	CCL2	14.58	9.02	131.59	Secreted	Immune
interleukin 1, beta	IL1B	14.44	0.82	11.80	Secreted	Immume
chemokine (C-X-C motif) ligand 3	CXCL3	14.00	2.72	38.15	Secreted	Immume
intercellular adhesion molecule 1 (CD54), human rhinovirus receptor	ICAM1	13.88	1.06	14.69	TM	Immume
tryptophan hydroxylase 1 (tryptophan 5-monooxygenase)	TPH1	13.37	2.91	38.95	Intracellular	Enzyme
CD274 molecule	CD274	12.43	0.90	11.16	TM	Immune
solute carrier family 25 (mitochondrial carrier), member 18	SLC25A18	11.33	0.92	10.38	Mitochondrial	Carrier
sema domain, immunoglobulin domain (Ig), short basic domain, sec, ted, (semaphorin) 3D	SEMA3D	11.18	13.44	150.30	TM	Stem cell
Transcribed locus	Hs.163426	11.07	1.27	14.07	N/A	Non-coding
insulin-like growth factor binding protein 3	IGFBP3	10.21	2.27	23.22	Intracellular	Unknown
dual specificity phosphatase 4	DUSP4	9.40	2.78	26.14	Intracellular	Signaling
pleckstrin homology-like domain, family B, member 2	PHLDB2	7.90	2.36	18.63	Intracellular	Structural
chromosome 16 open reading frame 54	C16orf54	7.58	3.16	23.95	TM	Unknown
ephrin-B3	EFNB3	7.50	1.41	10.57	TM	Receptor
synaptotagmin XIII	SYT13	7.22	15.90	114.69	Vesicular	neuronal
zinc finger, matrin type 4	ZMAT4	7.18	1.53	11.00	Nuclear	Transcription factor
chromodomain protein, Y-like 2	CDYL2	6.64	3.85	25.60	Nuclear	Metabolism
taste receptor, type 1, member 1	TAS1R1	6.08	1.95	11.84	TM	Taste
neuropilin (NRP) and tolloid (TLL)-like 2	NETO2	5.90	1.74	10.27	TM	Receptor
Jumonji, AT rich interactive domain 1A (RBBP2-like)	JARID1A	5.48	2.02	11.09	Nuclear	Transcription factor
fibroblast growth factor 19	FGF19	5.40	2.09	11.29	Secreted	Growth factor
FERM domain containing 4A	FRMD4A	5.37	3.30	17.72	Intracellular	Unknown

Genes with a FG versus circumvallate (CV) TB expression ratio ≥5-fold are listed. Expression ratios of CV or FG TB versus lingual epithelium (LE) samples are also included. Location; known or predicted location of gene product, TM; known to be membrane-associated or with a predicted transmembrane domain, N/A; not applicable. Function; known or predicted function of gene product.

### Functional Classification of Taste Genes

To better classify and organize the large list of genes identified, we annotated all genes in the database to assign a primary function and tabulated the results (Table S5). Five functional classes are highlighted, supporting trends identified by the analysis of the top 50 (Tables 2 and 3) and region-specific (Tables 4 and 5) lists.

#### Neuronal

We identified 90 neuronal-associated genes including many not previously associated with taste buds (Tables 2 and 3, Table S2). Expression of these genes is consistent with the primary function of taste buds to signal the presence of sweet, bitter, umami, sour, and salty tastants in saliva and transmit this information via nerve fibers to gustatory centers in the brain. Accordingly, genes encoding neurotransmitter receptors (n = 8) and synaptic vesicle/synapse proteins (n = 20) were highly represented. Neurotransmitter receptors include two adrenergic receptors, ADRA1A and ADRB1, the adenosine A2b receptor, ADORA2B, and a purinergic receptor, P2RX4, while SNAP25 [18], synapsin II (SYN2) [19], [20] and four synaptotagmins (SYT1, 4, 11 and 13) [21] are examples of synapse-associated gene products. We also identified genes encoding adhesion proteins that participate in neuronal cell interactions including NRCAM that had not previously been associated with taste buds. Other adhesion proteins define specific cell types in the taste bud, notably NCAM1 expressed by type III cells [22]. Furthermore, many genes associated with central nervous system development were represented (n = 15), underscoring both the neuronal nature and continuous turnover of taste bud cells. ASCL1, a basic helix-loop-helix transcription factor associated with developing neurons, is co-expressed with PROX1 in murine taste buds [23], [24], and both genes were expressed in macaque taste buds.

#### Immune

**Figure 2:**
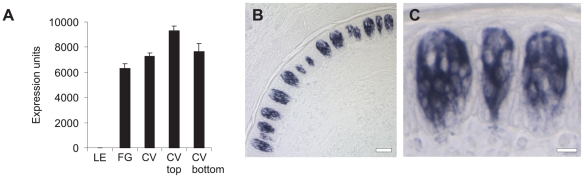
Expression of CXCL14 mRNA in macaque CV taste tissue. (A) Mean microarray expression values±SEM for CXCL14. (B) *in situ* hybridization showing CXCL14 expression in CV taste buds. Scale bar is 30 µm. (C) Zoom of CV taste buds expressing CXCL14. Scale bar is 10 µm.

Identification of a large group of genes associated with the immune system (n = 43) was both interesting and surprising. Multiple chemokine and cytokine genes are expressed in taste buds. CXCL14 represents the most highly expressed TB-associated gene (Figure 2A). CXCL14 expression was confirmed using qPCR with RNA isolated from human taste buds (data not shown) and also by *in situ* hybridization in macaque CV papillae (Figure 2B-C) which shows that CXCL14 is readily detected in many macaque taste bud cells but absent in adjacent lingual epithelium. Genes encoding innate immunity-associated proteins also feature prominently in this functional class including several members of the complement system, C20orf114 (a member of the PLUNC family of host defense proteins) and toll-like receptor 1 (TLR1).

#### Sensory

Genes in this functional class (n = 27) were defined as those expressed at sensory sites distinct from the taste bud including the olfactory epithelium (n = 4), ear (n = 4), eye (n = 13), and multiple sensory sites (n = 6). SLIT and NTRK-like family, member 6 (SLITRK6), is expressed at multiple sensory sites (including otic cyst, pharyngeal arches, cochlea, retina and tongue) during mouse development in conjunction with leucine rich repeat neuronal 3 (LRRN3) [25], that is also highly expressed in taste buds (Table S2). Genes associated with the olfactory epithelium include contactin 4 (CNTN4), Kallmann syndrome 1 sequence (KAL1), and olfactomedin 2 (OLFM2); genes associated with the ear include espin (ESPN), sine oculis homeobox homolog 1 (SIX1), and deafness, autosomal recessive 59 (DFNB59); and genes associated with the eye include eyes absent homolog 1 (EYA1), sidekick homolog 2 (SDK2), and dachshund homolog 1 (DACH1). Several of these, including KAL1, DFNB59 and EYA1 are associated with human genetic disorders that lead to sensory defects [26]-[28].

**Figure 3:**
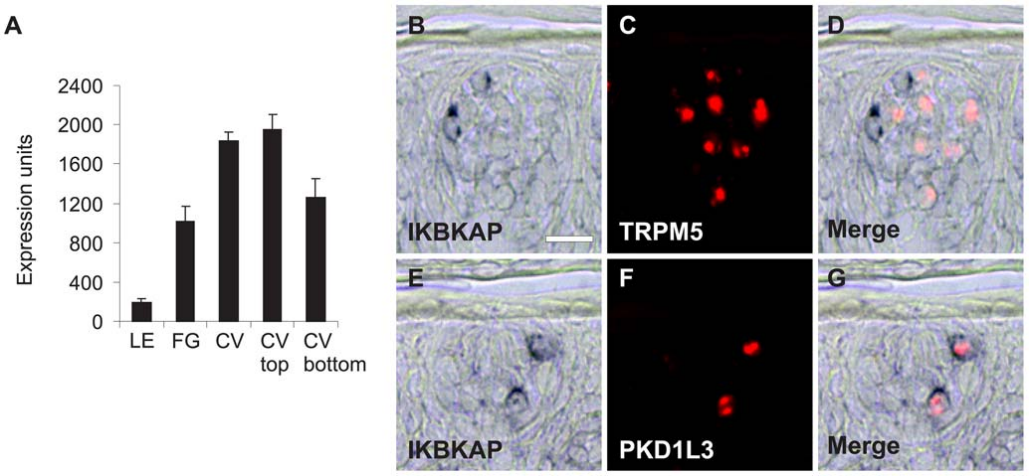
Expression of IKBKAP mRNA in macaque taste tissue. (A) Mean microarray expression values±SEM for IKBKAP. (B–G) *in situ* hybridization showing IKBKAP expression in PKD1L3 cells in CV taste buds. IKBKAP expression was visualized using colorimetric detection (purple color, left panels). Taste genes (TRPM5 and PKD1L3) were visualized using fluorescent detection (red color; center panels). Merged images (right panels) show signals from IKBKAP and taste genes. (B) IKBKAP, (C), TRPM5 (marker of sweet, bitter, and umami cells), and (D) merge showing expression of IKBKAP and TRPM5 in different cells. (E) IKBKAP, (F), PKD1L3 (sour cell marker), and (G) merge showing expression of IKBKAP in PKD1L3 cells. Scale bar is 15 µm in B and represents panel B–G.

One additional member of the sensory gene class encodes the inhibitor of kappa light polypeptide gene enhancer in B-cells, kinase complex-associated protein (IKBKAP) (Figure 3A). Mutations in IKBKAP cause familial dysautonomia [29], [30], a disease resulting in sensory and autonomic neuropathies characterized by loss of taste buds and nerves innervating taste buds [31], [32]. Using double label *in situ* hybridization, IKBKAP was found selectively expressed in taste cells that express PKD1L3 in macaque CV taste buds (Figure 3B-G).

#### Taste bud development

**Figure 4:**
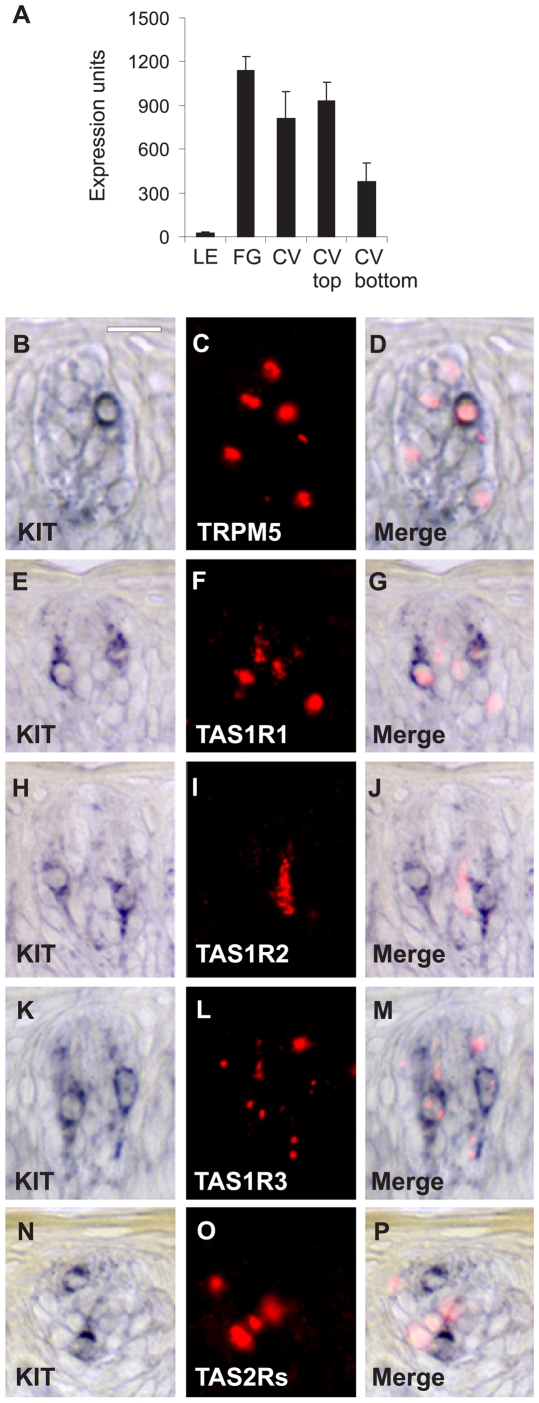
Expression of KIT mRNA in macaque taste tissue. (A) Mean microarray expression values±SEM for KIT. (B–P) *in situ* hybridization showing KIT expression in TAS1R1 cells in CV taste buds. KIT expression was visualized using colorimetric detection (purple color, left panels). Taste genes (TRPM5) and taste receptors (TAS1R1, TAS1R2, TAS1R3, and TAS2Rs) were visualized using fluorescent detection (red color; center panels). Merged images (right panels) show signals from KIT and taste genes. (B) KIT, (C), TRPM5 (marker of sweet, bitter, and umami cells), and (D) merge showing coexpression of KIT in a subset of TRPM5 cells. (E) KIT, (F), TAS1R1 (umami receptor), and (G) merge showing expression of KIT in a subset of TAS1R1 cells. KIT was expressed in approximately half of TAS1R1 cells. (H) KIT, (I) TAS1R2 (sweet receptor), and (J) merge showing expression of KIT and TAS1R2 in different cells. (K) KIT, (L) TAS1R3 (sweet and umami co-receptor), and (M) merge showing expression of KIT in a subset of TAS1R3 cells (these cells would also express TAS1R1). (N) KIT, (O) TAS2Rs (bitter receptors), and (P) merge showing expression of KIT and TAS2Rs in different cells. Scale bar is 15 µm in B and represents panels B–P.

Unlike sensory cells in the inner ear and retina, taste bud cells are in a constant state of renewal and turnover every ten to fourteen days [33], [34]. The dynamic nature of taste buds is illustrated by the expression of genes associated with stem cells (n = 15), growth factors (n = 28), receptors (n = 43), and development (n = 30). Included are receptor-ligand pairs such as sonic hedgehog (SHH) and patched homolog 1 (PTCH1) as well as v-kit Hardy-Zuckerman 4 feline sarcoma viral oncogene homolog (KIT) (Figure 4A) and KIT ligand (KITLG). Using double label *in situ* hybridization, we determined that KIT is expressed in a subset of TRPM5 cells (encompassing sweet, bitter, and umami taste cells) (Figure 4B–D). By labeling with TAS1R1 (umami receptor), TAS1R2 (sweet receptor), and TAS2R (bitter receptors) probes, we observed that KIT is selectively expressed in a subset of TAS1R1 taste cells that also express the umami co-receptor TAS1R3 (Figure 4E–P).

#### Endocrine

Genes encoding both peptide hormones (n = 7) and hormone receptors (n = 5) are abundantly expressed in taste buds. In some cases, ligand/receptor pairs such as growth hormone releasing hormone and its receptor (GHRH/GHRHR) and growth hormone 1 and its receptor (GH1/GHR) are co-expressed and exhibit similar expression profiles. Taste buds also express proopiomelanocortin (POMC, the precursor of melanocortin), parathyroid hormone-like hormone (PTHLH), and oxytocin (OXT) as well as receptors for insulin (INSR) and secretin (SCTR). Other endocrine-associated genes encode either receptors (NPR2, SSTR1) or ligands (STC2, RARRES2).

## Discussion

We used a systematic approach to generate a high quality database of primate taste bud gene expression. The database represents the first genome-wide survey of gene expression in taste buds from a primate (cynomolgus macaque) and is a significant milestone in defining the molecular components underlying the processes of taste. The limited abundance and difficulty in isolating primate taste buds devoid of contaminating lingual epithelial cells that has hindered molecular analysis of primate taste cell gene expression was overcome by using the macaque as a source of tissue and LCM as the method of tissue collection. Two additional factors enabled accurate, sensitive and comprehensive transcriptional profiling of primate taste buds: rapid tissue collection resulting in minimal post-mortem RNA degradation, and the recent development of a genome-wide microarray specific for the macaque.

Our results demonstrate the power of transcriptional profiling of microdissected tissue to better understand the components and pathways active in that tissue. While these techniques have previously been applied primarily to diseased and pathogenic tissues [35], [36], we believe that transcriptional profiling of LCM samples from normal tissue holds great promise to catalog the molecular components responsible for specialized tissue functions. Comprehensive analysis of taste bud gene expression has allowed us to make multiple specific and significant observations regarding the makeup and function of taste buds.

Spatial patterns of gene expression.

We observed region-preferential expression of taste receptors in macaque taste buds. TAS1R1 was expressed at higher levels in FG TB and T2Rs were expressed at higher levels in CV TB, similar to rodents [37], [38]. Slightly higher expression of TAS1R2 in FG TB compared to CV TB in macaques may indicate species differences since this gene exhibits lower expression in FG TB in rodents [38]. Enrichment of mRNA for taste receptors toward the top of taste buds is consistent with apical expression of taste receptors that facilitates access to tastants in saliva at the taste pore region. This observation suggests that transcripts corresponding to potential novel taste receptors, receptor-associated proteins, and signal transduction molecules will also be enriched in the top fraction of taste buds. For example, we observed that G protein-coupled receptor 113 [39] transcripts are enriched in the top fraction of taste buds and present in a population of taste cells that express TRPM5 but not sweet or bitter taste receptors. Similarly, we observed that MCOLN3 [40] is expressed in a discrete cell population in taste buds (data not shown).

### Taste bud development and turnover

Our data has revealed the dynamic nature of taste buds. During development, taste buds arise from epithelium in a complex process involving a variety of factors including SHH, PTCH1, bone morphogenic proteins and neurotrophic factors [12], [41], [42]. Members of the frizzled receptor and wingless-type MMTV integration site families have been implicated in cell turnover [43], and their expression has been reported in taste buds [44]. Many of these genes and their close relatives are present in the database (SHH, PTCH1, DISP1, BDNF, BMPR1B, NOTCH4, FZD1, 3 and 4, WNT5A) or are expressed at high levels in macaque taste buds (NDP, WNT5B, WNT11). These receptor-ligand pairs may act within taste buds in a paracrine and/or autocrine manner to generate diverse taste cell types. Both SHH and PTCH1 are expressed in taste tissue during embryogenesis [12], [45]. In adult taste buds, expression of SHH is associated with basal cells that differentiate into mature taste receptor cells [3]. The identification of multiple members of both the SOX and HOX gene families (HOXA3, A10, B2, B3, and SOX1, 2, 4, 5, 21) indicates that taste bud renewal utilizes pathways active in taste bud development. Correlating early developmental processes with ongoing taste bud renewal may help elucidate mechanisms of taste cell turnover.

The tyrosine kinase receptor KIT controls stem cell survival and development in bone marrow, skin, and gut [46]. KIT is expressed in both developing and adult taste buds in the rat [47], and our data shows that both KIT and its ligand are expressed in primate taste buds pointing to the presence of taste progenitor cells. However, the specific cells that express KIT had not been previously established. Our *in situ* hybridization data indicate that KIT is expressed exclusively in TAS1R1 (umami) cells. While the functional role of KIT in taste cells has yet to be determined, KIT may modulate umami cell differentiation and development. Indeed, patients treated with the KIT antagonist imatinib mesylate (Gleevec) reported alterations in taste perception [48], [49]. To our knowledge, the mechanism for the altered taste side effect of this compound is currently unknown. Our results provide a potential explanation for this observation and a direct role for KIT in taste.

Mutations in IKBKAP cause familial dysautonomia, a disease that leads to loss of taste buds and nerve fibers innervating taste buds [31], [32]. We have demonstrated that IKBKAP is selectively expressed in sour (type III) cells in macaque taste buds. These cells form direct synapses with afferent nerve fibers. The IKBKAP gene product has recently been shown to participate in actin fiber organization and cell migration [50], suggesting that this cell population may require IKBKAP to migrate and that absence of this function may affect taste bud survival. Alternatively, since this taste cell population releases neurotransmitters by exocytosis onto afferent nerve fibers, and since the yeast homolog of IKBKAP, Elp1p, interacts with the post-Golgi vesicular transport component Sec2p [51], IKBKAP may regulate polarized exocytosis of signaling components in sour taste cells. Absence of these and other signaling pathways in taste buds and nerve fibers in individuals with IKBKAP mutations may impact taste bud survival.

KIT and IKBKAP are representative of many genes we found expressed in taste cells dedicated to distinct taste modalities. This finding supports the labeled line model of taste coding in the periphery [4], where different taste cells, defined by what specific taste receptor they express, represent different lineages with specific gene expression patterns.

In addition to factors expressed within taste cell progenitors, taste buds require nerve innervation to survive. In this context, the presence of several genes associated with axonal growth and guidance including semaphorins (SEMA4C, SEMA5A) [52], [53] and protein tyrosine phosphatase receptors (especially type D and S) [54], may generate the foundation for a labeled line system whereby nerve fibers transmitting responses for specific taste modalities selectively innervate taste cells expressing receptors for those modalities. Indeed, protein tyrosine phosphatase receptor type S participates in neuronal development of the retina, a site of sensory nerve fiber growth [55].

Despite originating from epithelium, taste buds express genes associated with neurons and central nervous system development, maintenance, and function. The achaete-scute complex-like 1 (ASCL1 or MASH1) is highly expressed in primate taste buds and participates in commitment and differentiation of distinct neuronal cell types including sensory neurons [56], [57]. Several neuron-specific adhesion molecules are expressed including neuronal cell adhesion molecule (NRCAM), L1 cell adhesion molecule (L1CAM) and integrin, alpha 3 (ITGA3) not previously identified in taste buds. These molecules may function in subsets of taste cells to modulate specific cell-cell interactions required for a labeled line signal transmission paradigm.

### Taste buds may play an active role in protection from pathogens

Several genes associated with the immune system are present in the taste bud database. We did not detect significant expression of genes encoding leukocyte markers such as immunoglobulins, T cell receptor-associated, or myelomonocytic-associated proteins in taste buds. Furthermore, we did not observe leukocyte infiltrates in taste bud sections, implicating taste cells as the origin of immune gene transcripts. Also, genes were selected because they were expressed in taste buds and not significantly expressed in adjacent LE. The taste bud represents a potentially favorable site for pathogen entry via the taste pore. Interestingly, the chemokine CXCL14 is the highest expressed taste-bud associated gene in our database. While the receptor for this chemokine is unknown, it is broadly expressed in adult tissues (breast, kidney) and has been reported to be a chemoattractant for dendritic cells and monocytes [58], [59], although mice lacking this chemokine do not exhibit deficiencies in dendritic cell trafficking [60]. However, we did not observe monocytes or detect monocyte-associated gene expression in taste buds. CXCL14 may be secreted across the apical membrane of taste bud cells to become a component of saliva. Leukocytes have been found in human saliva [61], and the focal release of chemokines may attract immune cells to the taste pore. Another chemokine, CXCL12 and its receptors CXCR4 and CXCR7 participate in neuronal survival in multiple areas of the brain [62], [63]. Furthermore, CXCL14 defective mice exhibit metabolic defects [64] and may have abnormal taste buds implying that CXCL14 could play a direct role in the development or regulation of taste buds.

Expression of complement components (C1R, C2, and C3) may also represent chemotactic signals to the taste pore. Local production of these factors as well as cytokine receptors of the interferon-signaling cascade by taste bud cells [65] may contribute to innate immunity.

### Taste bud/endocrine system link

Expression of hormone receptor and ligand transcripts, normally associated with endocrine glands, within taste buds adds to a growing body of evidence for a cephalic response to feeding. It is well known that plasma insulin levels rise rapidly following ingestion of a carbohydrate rich meal and prior to the subsequent rise in plasma glucose levels [66], [67]. What is less clear is what portion of the cephalic response is anticipatory (centrally-mediated) versus direct (peripherally-mediated) [68]. We have shown that macaque taste buds express proopiomelanocortin (POMC), growth hormone releasing hormone (GHRH), parathyroid hormone-like hormone (PTHLH) and oxytocin (OXT) and the receptors for insulin (INSR), growth hormone (GHR), growth hormone releasing hormone (GHRHR) and secretin (SCTR). POMC is the precursor of melanocortin, the ligand of the MC4 receptor which plays an important role in feeding behavior [69]. Taste buds may communicate with the gut via release of these endocrine/neuroendocrine hormones. Expression of hormone receptors suggests a feedback mechanism by which taste sensation is modulated according to nutritional status. Shin et al recently reported that sweet taste is regulated by GLP-1 in a paracrine mechanism whereby GLP-1 released by taste cells binds receptors on adjacent nerve fibers to modulate sweet taste responses [70]. Endocrine-associated gene products in taste buds may function in the cephalic response to feeding that prepares the gut for digestion of a meal [68]. The role played by peptide hormones generated within taste buds in the cephalic response requires further study.

Our database of taste bud gene expression will open new lines of investigation and lead to a better understanding of taste bud physiology in normal and diseased states. Of considerable interest are the 349 TB-associated genes in the database with unknown function. Analysis of these and other functional classes will reveal additional interesting pathways and processes active in taste buds. We have confirmed the expression of most genes of interest described in this study in human taste buds isolated by LCM, thereby confirming the macaque gene array findings. In addition, we are currently mapping more genes to specific taste cell populations and have identified new markers of known taste cell types as well as markers that define new taste bud cell types. Some of these markers may potentially define additional types of cells that may mediate novel taste modalities. Because the database contains most TB-associated genes, genes for novel taste receptors should be represented. Indeed, we have used the database to identify novel, candidate taste receptors. The identification of growth factors and growth factor receptors in taste buds raises the possibility of developing therapies to increase the survival and replenishment of taste bud cells. Since taste sensation declines with age [71] and in patients undergoing therapy for head and neck cancers [72], [73], the availability of agents that may prevent taste loss in these groups would help maintain a nutritious diet and promote healthy outcomes.

## Materials and Methods

### Macaque and Human Samples

All primate samples were collected in compliance with applicable federal, state, and local laws and regulations (CFR 1985 and PHS 1996) according to IACUC recommendations and oversight at both Charles River and Covance. All human samples were collected with full written consent and with the approval of the Zoion Diagnostics institutional review board (IRB), an external independent IRB and the IRB or Human Studies Committee (HSC) at the organizations directly involved with the collection for final approval. Taste tissue from cynomolgus macaques (*Macaca fascicularis*; 2.6–4.5 years old; males and females) was collected post-mortem by Charles River (Sparks, NV) and Covance (Alice, TX) from animals scheduled for euthanasia for other purposes. Tissue was obtained with a post-mortem interval of 10 minutes, embedded in OCT freezing medium (Triangle Biomedical Science, Durham, NC), and frozen in liquid nitrogen. Human tongue samples were purchased from Zoion Diagnostics (Hawthorne, NY) as OCT embedded blocks and stored at −80°C. Human taste tissue was obtained from Caucasian post-mortem donors less than 30 years of age, who were non-smokers with no known alcohol or drug use, with a post-mortem interval less than 5 hours.

### RNA Extraction and Gene Expression Analysis

LCM was used to isolate taste tissue from macaque and human samples. Tissue sections (10–12 um thick) were cut on a Leica CM1850 cryostat, collected on RNase-free membrane slides (Molecular Machines and Industries, MMI, Rockledge, FL), and stained with cresyl violet using the Ambion LCM staining kit (Austin, TX) as per the manufacturer's instructions. Taste bud and lingual epithelial areas were isolated using a MMI Cellcut laser microdissection system on an Olympus IX71 inverted microscope and collected on MMI reaction tube adhesive lids. For macaque, both CV and FG papillae were used; for human, only CV papillae were used. Material from multiple sections was pooled, and all LCM samples were collected within 2 hr of sectioning. One paired TB pool and LE pool were collected per donor from multiple donors. Following collection, total RNA from taste bud and lingual areas was separately purified using a Qiagen microRNeasy kit (Valencia, CA) and evaluated using an Agilent 2100 Bioanalyzer with a Series II RNA 6000 Pico Assay. Total RNA was amplified to generate cDNA using the WT Ovation Pico system (NuGEN, San Carlos, CA). Microarray gene expression data was generated from the macaque samples by Gene Logic using Affymetrix GeneChip® Rhesus Macaque Genome Arrays (Affymetrix, Santa Clara CA). All microarray experiments in this study were done in accordance with MIAME guidelines and the gene expression data sets presented have been deposited in the GEO database (GEO series entry GSE16485). Semi-quantitative PCR expression data was generated from amplified human cDNA samples using the Mx3000P® QPCR System (Stratagene, La Jolla CA) and inventoried TaqMan assays (Applied Biosystems, Foster City, CA).

### Selection and Analysis of Taste Bud-specific Genes

Microarray data was analyzed using GeneSpring GX software (Agilent Technologies, Santa Clara, CA). Raw data from the Rhesus Macaque Genome Arrays were processed using GC-RMA normalization [9]. FG TB, CV TB and LE data were compared by using two-sample paired *t*-tests. Genes were defined as TB-associated if they met the following inclusion criteria: minimum mean TB expression ≥25, fold expression difference (TB versus LE) ≥5 with a p value ≤0.05. Genes were selected from each set of pairwise comparisons to give 4 lists (CV versus LE, FG versus LE, CV bottom versus LE and CV top versus LE) that were subsequently combined to give a single non-redundant master list.

### *in situ* hybridization

Fresh frozen sections (10–12 µm thick) were attached to RNase-free SuperFrost Plus slides (Fisher Scientific, Pittsburg, PA) and processed for in situ hybridization as described [74]. Because human and macaque genomes are ∼95% homologous [6], we hybridized human riboprobes to macaque tissue. Riboprobes were generated for TRPM5 (NM_014555; nt 396-2,006), PKD1L3 (NM_181536; nt 1-1,079), TAS1R1 (NM_138697; nt 1,103-2,526), TAS1R2 (NM_152232; nt 2-1,002), TAS1R3 (NM_152228; nt 1-1,311), a pool of 6 TAS2Rs – TAS2R8 (NM_023918; nt 1-930), TAS2R10 (NM_023921; nt 1-924), TAS2R13 (NM_023920; nt 265-1,176), TAS2R14 (NM_023922; nt 1–954), TAS2R48 (NM_176888; nt 1-900), and TAS2R50 (NM_176890; nt 53-952), IKBKAP (NM_003640; nt 2087-3621), KIT (NM_000222; nt 102-1649), and CXCL14 (NM_004887 559 to 1794). Digoxigenin and fluorescein labeled riboprobes were used to detect expression of two different genes in taste bud cells. Signals were developed a using colorimetric-fluorescent detection method. For colorimetric-fluorescent detection, fluorescein-labeled riboprobes were first developed with peroxidase-conjugated anti-fluorescein antibody with tyramide signal amplification (TSA)-Cy3 (Perkin Elmer, Waltham, MA) and digoxigenin-labeled riboprobes were subsequently developed with alkaline phosphataste-conjugated anti-digoxigenin antibody (Roche, Indianapolis, IN) with NBT/BCIP substrate. Control hybridizations with sense riboprobes demonstrated signal specificity. Specimens were viewed on an Eclipse E600 upright microscope (Nikon Instruments, Inc., Melville, New York) equipped with a Plan Fluor 20× objective and a 100 W mercury arc lamp. Fluorescence associated with TSA-Cy3 was collected using the following filter sets: 528-553 excitation, 565 dichroic beamsplitter, and 600–660 emission. Images were acquired using the Spot RT System and Spot v4.6.4.6 software (Diagnostic Instruments, Inc., Sterling Heights, MI), saved in TIFF format, and processed using Adobe Photoshop v9.0.

## Supporting Information

Figure S1 Principal component analysis of microarray data.
(7.92 MB TIF)

Table S1 Microarray sample information.
(0.02 MB XLS)

Table S2 Macaque taste bud gene expression database of 2,382 genes.
(0.74 MB XLS)

Tabe S3 Genes associated with the bottom fraction of circumvallate taste buds.
(0.03 MB XLS)

Table S4 Genes associated with the top fraction of circumvallate taste buds.
(0.03 MB XLS)

Table S5 Representation of 2,382 taste bud-associated genes by functional classification.
(0.02 MB XLS)
