# Supplementary figures and images for: Genome-Wide Analysis of Gene Expression in Primate Taste Buds Reveals Links to Diverse Processes
Source: PLoS One. 2009 Jul 28;4(7):e6395. doi: 10.1371/journal.pone.0006395 (PMC2712080; doi:10.1371/journal.pone.0006395)

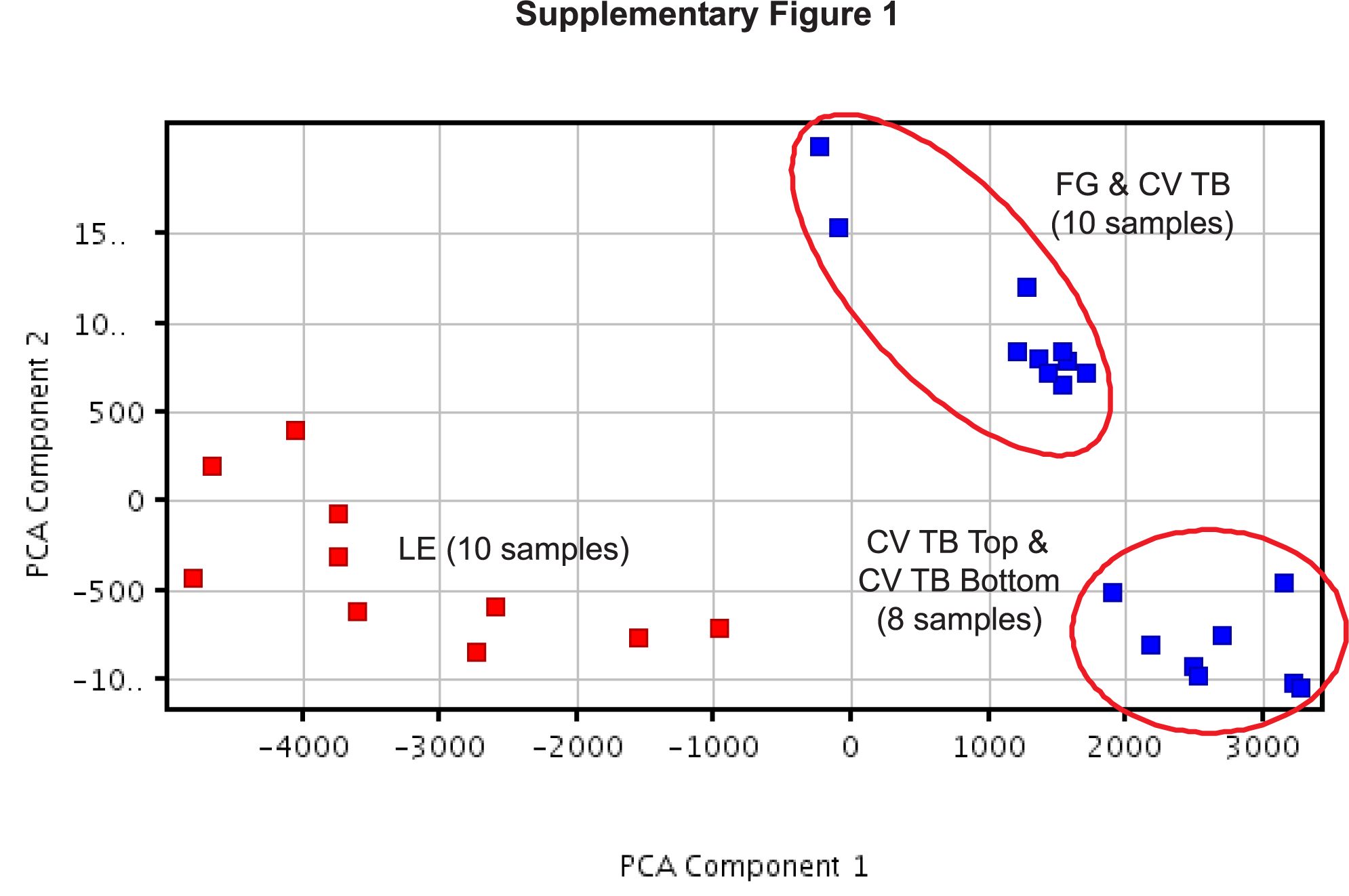

Supplement: Figure S1 — Principal component analysis of microarray data. (7.92 MB TIF) [file pone.0006395.s001.tif]
